# Artist profiling using micro-CT scanning of a Rijksmuseum terracotta sculpture

**DOI:** 10.1126/sciadv.adg6073

**Published:** 2023-09-20

**Authors:** Dzemila Sero, Isabelle Garachon, Erma Hermens, Kees Joost Batenburg

**Affiliations:** ^1^ Centrum Wiskunde & Informatica, Science Park 123, Amsterdam 1098 XG, Netherlands.; ^2^ Conservation & Science, Rijksmuseum, Hobbemastraat 22, Amsterdam 1071 ZC, Netherlands.; ^3^ Hamilton Kerr Institute & Conservation and Science Division, Fitzwilliam Museum, University of Cambridge, Cambridge, UK.; ^4^ Leiden Institute of Advanced Computer Science, Niels Bohrweg 1, Leiden 2333 CA, Netherlands.

## Abstract

In the fine arts, impressions found on terracotta sculptures in museum collections are scarcely reported and not in a systematic manner. Here, we present a procedure for scanning fingermarks and toolmarks found on the visible surface and inner walls of a terracotta sculpture using 3D micro–computed tomography, as well as methods for quantitatively characterizing these impressions. We apply our pipeline on the terracotta sculpture *Study for a Hovering Putto*, attributed to Laurent Delvaux and housed in the Rijksmuseum collection. On the basis of combined archaeology and forensics research that assigns age groups to makers of European ancestry from ridge breadth values, we estimate that the fingermarks belong to an adult male. Given that each fingerprint is unique and the carving tools were exclusively made in the artist’s workshop, we give incentive to aim for artist profiling using innovative computational approaches on preserved impressions from terracotta sculptures.

## Teaser

A pipeline to acquire and analyze preserved fingermarks and toolmarks is implemented using 3D micro Computed Tomography.

## INTRODUCTION

In cultural heritage, preserved fingermarks are finger ridge impressions of past makers left on soft and adhesive materials. Wet, soft clay is malleable and records every impression. When fired, clay becomes a hard and water-resistant solid known as “terracotta,” an Italian word meaning “clay that has been fired.” Hence, the majority of preserved fingermarks have been found on clay (*1*), which was used by artisans from various societies to create everyday items such as tableware, cooking utensils, vessels, figurines, and tiles. In ideal conditions, the impressions on clay can be preserved for centuries. However, external factors affecting the surface during deposition and postdeposition, such as mishaps, surface polishing and glazing, shrinkage cracks, environmental effects (soluble salts, weathering, and biological deterioration), and poorly executed restorations, can obscure these impressions. Usually, surviving fingermarks are only partial because they are unintentional marks from the manufacturing process. Environmental conditions, as well as handling, may cause additional damage or erasure.

Each fingerprint features a distinct and detectable pattern of alternating ridges and furrows. As a result, fingerprints are the most widely used biometric identifiers and are used in situations requiring authentication/verification, such as international border controls, financial fraud detection, cyber security, smartphone locking systems, and e-commerce and e-governance applications (*2*). Patterns from a fingermark of an unknown person are compared against patterns from fingerprints of known individuals stored in a database, and the result gives a probability score representing how well the fingerprints match each other. Matching is only possible if large portions of the fingermark are preserved and of sufficient quality. In pottery, given that preserved fingermarks are often incomplete and that a database of marks from a few known individuals is unavailable for most workshops outside one or two from the Mediterranean, finding matching impressions is rare (*3*). Furthermore, forensic experts are careful when comparing fragmentary or damaged impressions in archaeological finds as this may result both in false positives (two partial areas match even if they belong to two different fingermarks) and false negatives (two partial areas of the same fingermark display different papillary lines).

When traditional research on stylistic and technological variations falls short of improving the understanding of past human activity over thousands of years, the analysis of preserved fingermarks found on pottery constitutes an alternative method (*4*–*8*). Fingermarks are used to ascertain the demographics (sex and age) of the makers even when incomplete marks are provided and neither the finger nor the section of the finger they represent can be recognized. Since changes in ridge breadth and ridge density are mainly influenced by aging and sexual dimorphism (i.e., the typical differences in body shape and size between males and females), the potential of estimating the age and sex of past artisans by analyzing finger ridges has rekindled interest in fingermark identification in archaeology. In particular, during a practical experiment where volunteers leave fingerprints on plasticine (*6*), scholars observe a high correlation between the volunteers’ age and ridge breadth. Experts then determine linear regression models to estimate the age category of past makers (*4*, *6*), and Fowler *et al.* (*5*) propose three age groupings: children, adults/adolescents, and adults. To determine sex, specialists estimate the number of ridges in either 25 or 6 mm^2^ of area (*9*).

During archaeological investigations, experts bring high-resolution and portable cameras with lenses suitable for high magnification macrophotography. The resulting photographs are uploaded to local or remote computers, and commercial software is then used for image calibration and contrast enhancement. In bidimensional (2D) images, the appearance of an epidermal ridge imprinted on ceramics is caused by the contrast between light and shadows cast from an artificial light source. Because most impressions found on pottery are incomplete and, to some extent, deteriorated, it is challenging to capture these in difficult-to-reach spots. Digital calipers are also used, but measurements are greatly affected by human errors. Curvature is also an issue as marks or sections of marks on sharply curved surfaces cannot be measured accurately from 2D images.

In fine arts like sculpture, clay is often used by sculptors to make preliminary models, to make a sketch in clay. Therefore, clay models frequently show marks of hands and tools. In a few sculptures from Bernini, epidermal ridge impressions are exceptionally well preserved and matching impressions has been feasible using forensic expertise (*10*). Conservators usually use high-resolution 2D imaging technology in their diagnostic methods, such as camera-equipped stereo microscopes in museum conservation departments. However, there is no standard methodology. As fingermarks are frequently found in inaccessible locations, specialists have to adjust artificial lighting and select the optimal position of the artifact, resulting in a relatively long and tedious procedure. 3D imaging helps experts in museum settings with diagnostics, documentation, object decay assessment, texture visualization, digitization, and data interpretation. In particular, computed tomography (CT) and magnetic resonance imaging (MRI) are the most useful tools for full-volume inspection, especially for difficult-to-reach areas and internal voids.

The study of labor organization in potting communities of various societies contributes substantially to the development of methodologies to identify, collect, and analyze fingermarks found on the surface of archaeological ceramics (*5*–*8*, *8*, *11*–*13*). The seminal work of Kamp *et al.* (*6*) aims to identify the role of children in the manufacture of Ancient Puebloan ceramic vessels and figurines based on fingermarks impressed on these artifacts. Králík and Novotný (*4*, *14*) explore novel approaches to collect and study fingermarks left on clay artifacts. In another studies, Stinson (*12*) and Bennison-Chapman and Hager (*11*) estimate the demographics of makers from fingermarks found on ancient Hohokam artifacts and clay “tokens” from Neolithic West Asia, respectively. More recently, the work of Kantner *et al.* (*8*) aims to determine the sex from fingermarks found on pottery from the Ancestral Puebloan community in the American Southwest. Fowler *et al.* (*5*, *7*) implement methods of age and sex determination from fingermarks on sherds at Tell eṣ-Ṣâfi/Gath in Israel. Despite the renewed interest in preserved fingermarks in archaeology, research into 3D imaging technologies and computational methods for investigating fingermarks and toolmarks on sculptures has lagged behind (*1*). The primary reason is that capturing fingermarks on large amounts of archaeological finds with 2D portable cameras is a simpler and faster approach than with 3D volumetric imaging for any quantitative population studies needed in paleodermatoglyphics. In general, volumetric imaging is better suited to acquire images of single rare and fragile artworks from museums and private collections. Second, acquisition settings are linked with research objectives which are frequently oriented toward documentation or archival work, rather than collecting the best segments of marks for dactylographic analyses. Museums are increasingly using 3D portable cameras for photogrammetry, but these are not usually adopted for fingermark imaging. Moreover, full-volume inspection using CT and MRI setups requires trained operators, official permissions, large storage capacity, long-term hardware, and software maintenance, as well as physical room for expensive scanners, which museum laboratories often lack and outsource to other parties (e.g., universities, research institutes, and companies).

**Fig. 1.**
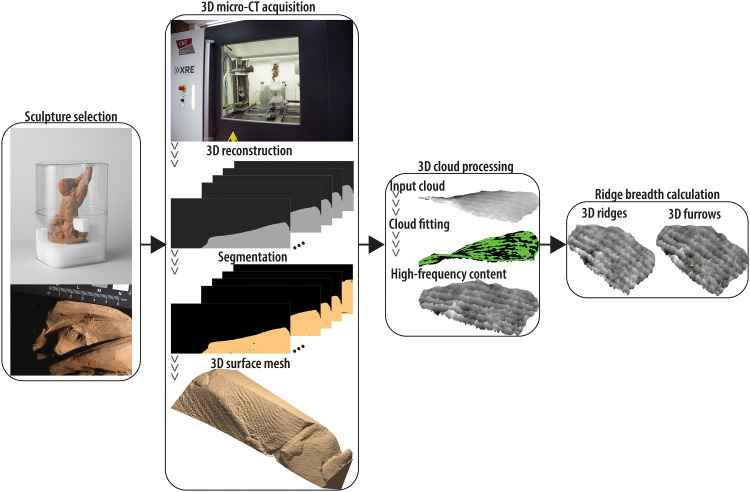
Schematic overview of our work. First, we select a sculpture from the Rijksmuseum collection. Second, we define CT motor positions for acquiring regions with impressions of interest (fingermarks and toolmarks). Following volume reconstruction, we convert the stack of slices with gray intensity values into a stack of binary images (segmentation) and generate a 3D surface mesh. Third, after fitting a surface to the original 3D data, we enhance the ridge/furrow content by subtracting the fitted data from the original shape. Last, we compute the breadth of ridges by drawing lines that intersect the ridges and furrows. Mean ridge breadth (*MRB)* is determined over three runs.

Here, we offer a noninvasive, reproducible methodology to image preserved fingermarks and toolmarks using 3D micro-CT technology (Fig. 1). As a case study, we apply our technology on a fired clay (or terracotta) sculpture from the Rijksmuseum permanent collection. Following a thorough inspection of terracotta artworks with the curator and conservator from the Rijksmuseum Department of Ceramics, Glass and Stone, we select the *Study for a Hovering Putto* (Rijksmuseum, BK-NM-9352), dated between 1735 and 1750, and attributed to Laurent Delvaux (Gent, 17 January 1696 and Nivelles, 24 February 1778). We study eight partial fingermarks on the visible surface and five in inner voids. Following volume reconstruction, we enhance the ridge/furrow alternations of each 3D impression to compute ridge count and breadth in a given area. Using regression models based on data from volunteers of European ancestry reported in the literature, we estimate the age clusters based on the ridge breadth. Using reference values from literature, our results indicate an adult male maker. Moreover, we study impressions from three clay carving tools, namely, three toothed modeling tools with teeth of varying width. Given that it was common practice in the 18th century to customize each carving tool on the artist’s requests (*15*), the corresponding toolmarks represent distinguishing traits of the artist and workshop practices. In addition, we conduct two experiments using commercial clay and tools to compare certain types of impressions left on fresh clay to those found on the sculpture’s surface. In one experiment, we ask a trained professional and an untrained volunteer to leave their fingerprints on fresh clay pieces: We ask the experienced ceramicist to leave fingerprints arbitrarily and then to fire the object, while the untrained participant leaves fingerprints with extreme care and does not fire the object. We CT scan both clay pieces for three main reasons. First, we demonstrate that our acquisition setup is also valid for objects made of either fresh or fired clay. Second, we show variation in ridge breadth values depending on the fingerprint section. Third, on the basis of age estimates on both samples, we confirm that these come from biologically adult makers when the same methods carried out on the Rijksmuseum sculpture are used. In the second experiment, we impress carving tools on fresh clay and compare these to strokes found on the artwork. Specifically, we show that thin linear striations found on the sculpture are most likely created by a brush with thin hair or a toothed modeling tool with narrow teeth. In general, the rationale for developing an interpretive framework for 3D impressions on clay sculptures using CT and image processing is presented. When combined with technical, stylistic, and art historical information, our analysis of fingermarks and handmade modeling tools could make it easier to discern papillary ridges from toolmarks, especially for partial sections where only a small segment of the total epidermal mark is impressed or preserved, thereby adding a sound criterion to characterize artist’s practice.

## RESULTS

### 3D reconstructions of preserved impressions and corresponding ridge breadth calculations

**Fig. 2.**
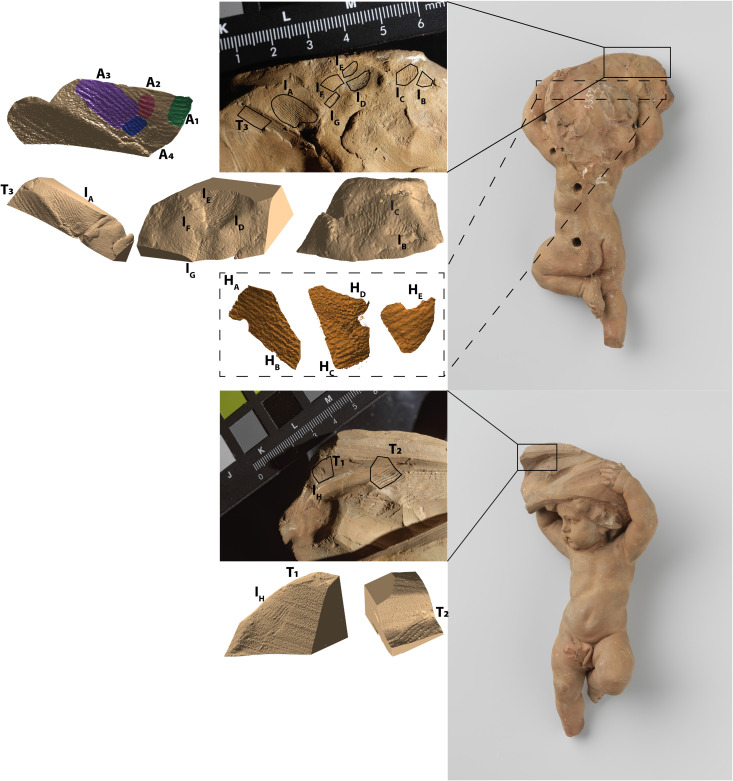
Schematic overview of the preserved impressions under investigation and their corresponding 3D CT-based reconstructions. Surface impressions are labeled as I_letter_ (I_A_, I_B_, I_C_, I_D_, I_E_, I_F_, I_G_, and I_H_), those found in internal voids hidden from view as H_letter_ (H_A_, H_B_, H_C_, H_D_, and H_E_), and toolmarks as T_number_ (T_1_, T_2_, and T_3_). The impression I_A_ is divided into four smaller sections (A_1_, A_2_, A_3_, and A_4_).

We study 16 impressions in total, including eight partial marks on the visible surface, five partial marks in inner voids completely hidden from view, and three strokes from clay carving tools (Fig. 2). We separately analyze four sections (A_1_, A_2_, A_3_, and A_4_) from the largest partial fingermark (I_A_).

**Fig. 3.**
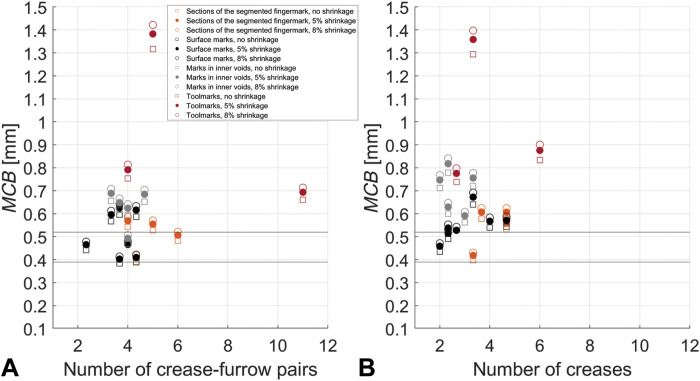
Distribution of MCB values corresponding to preserved impressions found on the Rijksmuseum terracotta sculpture. *MCB* values corresponding to each type of impressions (eight visible fingermarks, five fingermarks in internal voids, and three toolmarks) without shrinkage correction and with 5 and 8% shrinkage correction are plotted against the number of creases. Sections of the segmented fingermark refer to the four sections in I_A_. We determine each crease breadth value using the definitions of (**A**) Kamp and (**B**) Penrose. Horizontal lines at *MCB* = 0.39 mm and *MCB* = 0.52 mm refer to values found by Králík and Novotný (*4*) using fingerprints of European ancestry.

On each 3D-enhanced epidermal ridge impression and toolmark, we trace a straight line three times, resulting in three different measurements of ridge breadth. Multiple measurements refer to the same 3D model and do not include repeated CT scans of the object. For each single impression, mean crease breadth (*MCB*) refers to the average over three runs. Similarly, the mean crease-furrow count and the mean crease count refer to average values over three rounds. *MCB* values are then plotted against the mean crease-furrow count and the mean crease count (Fig. 3 and figs. S2 and S3). Table 1 shows *MCB* values for each impression, both values observed and corrected for shrinkage. Table S1 shows crease breadth values computed for each impression in each single run.

**Table 1.** *MCB* values corresponding to each preserved impression found on the Rijksmuseum terracotta sculpture. *MCB* values are expressed in mm. The total area (mm^2^) of each 3D impression is the sum of areas from single triangles composing the mesh. *MCB_O_* refers to the observed *MCB* and *MCB*_5%_ and *MCB*_8%_ account for 5 and 8% shrinkage, respectively [e.g., *MCB*_5%_ = *MCB_O_* + (*MCB_O_* × 0.05)].

Name	Area	Kamp			Penrose		
		*MCB_O_*	*MCB*_5%_	*MCB*_8%_	*MCB_O_*	*MCB*_5%_	*MCB*_8%_
A_1_	8.1	0.48	0.51	0.52	0.53	0.56	0.58
A_2_	4.9	0.54	0.57	0.59	0.58	0.61	0.62
A_3_	27.6	0.53	0.55	0.57	0.58	0.61	0.62
A_4_	4.8	0.39	0.41	0.42	0.40	0.42	0.43
I_B_	7	0.60	0.63	0.64	0.54	0.57	0.59
I_C_	22.4	0.59	0.62	0.63	0.64	0.67	0.69
I_D_	12.4	0.57	0.60	0.61	0.49	0.52	0.53
I_E_	6.4	0.44	0.47	0.48	0.44	0.46	0.47
I_F_	7	0.39	0.41	0.42	0.54	0.57	0.58
I_G_	8.9	0.38	0.40	0.41	0.51	0.54	0.55
I_H_	7.7	0.44	0.47	0.48	0.50	0.53	0.54
H_A_	10.4	0.59	0.62	0.64	0.78	0.82	0.84
H_B_	15.7	0.66	0.69	0.71	0.71	0.75	0.77
H_C_	15	0.65	0.69	0.70	0.72	0.76	0.78
H_D_	9.2	0.62	0.65	0.67	0.60	0.63	0.65
H_E_	10	0.47	0.49	0.51	0.56	0.59	0.61
T_1_	42.7	0.66	0.69	0.71	0.83	0.88	0.90
T_2_	42.5	1.32	1.38	1.42	1.29	1.36	1.40
T_3_	23.4	0.75	0.79	0.81	0.74	0.78	0.80

**Fig. 4.**
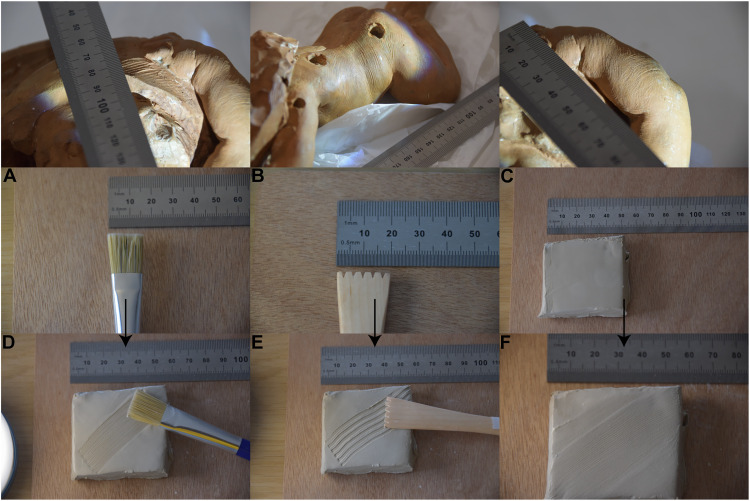
Qualitative experiments using modeling tools on wet clay. Top row: three areas of the sculpture displaying striations of different sizes. Center row: (**A**) paintbrush with dry, strong, and unused hair. (**B**) Toothed modeling tool with single tooth breadth of approximately 0.25 mm. (**C**) Piece of fresh and high fire clay. Bottom row: (**D**) brush strokes have dense creases, which are less deep than those left by the modeling tool. The differences in hair length justify the different depths of the striations. (**E**) The strokes on wet clay left by a toothed modeling tool are characterized by deep marks. (**F**) Smeared finger impressions on wet clay. Epidermal ridge impressions are almost imperceptible and more uniform in depth when compared to toolmarks.

*MCB* values determined using the definition of Kamp (Fig. 3A) are lower than those calculated with Penrose’s definition (Fig. 3B), and this connects with the way we calculate breadth values. In both cases, the origin and direction of the straight line are sometimes difficult to determine mainly because neither the start nor the end of a ridge/furrow is visible due to local deformation and clay irregularities. To ensure the best measurement, we place the origin and destination of the straight line between the peak and base of the first and last ridge/furrow of the bundle, respectively. Hence, the total distance between these two points is reduced, thus making the ratio between the distance and number of pairs lower, as seen in Fig. 3A. This also means that since Penrose’s definition considers ridge breadth as distance between centers of adjacent furrows, the resulting breadth is generally wider (Fig. 3B). In Fig. 3A, four visible partial fingermarks (I_E_, I_F_, I_G_, and I_H_), two sections from the largest visible fingermark (A_1_ and A_4_), and one partial fingermark in a void completely hidden from view (H_E_) fall in the group where 0.39 mm < *MCB* < 0.52 mm (*4*). In Fig. 3B, fingermarks I_D_, I_E_, and A_4_ fall within this range. The smallest *MCB* corresponds to the portion of the largest fingermark where clay is more deformed, I_E_, which we assume comes from a part of the fingertip that may have smaller ridges. Ridge breadth values are not indicative of a single or multiple fingermarks, nor which sections of a fingertip these values belong to. Since variation among ridge breadth values exists in different areas of the same fingermark, we run additional experiments to demonstrate this in controlled settings. On the basis of results collected in Fig. 3 and fig. S3, all carving tools have *MCB* > 0.7 mm even when shrinkage is not considered. Tool T_2_ has the largest *MCB* and corresponds most likely to a modeling tool with wide teeth, and T_1_ and T_3_ are strokes caused by modeling tools of varying width. Regarding fingermarks, the number of ridges, or number of ridge-furrow pairs in Kamp’s definition, is lower than six, which is expected given they are incomplete. The number of creases corresponding to carving tools varies depending on their underlying area. Here, ridge breadth values from fingermarks found in voids completely hidden from view are generally higher than those corresponding to surface marks. This result may suggest that clay was particularly wet in these regions, causing wider marks. We believe that these are human marks rather than toolmarks because pieces of clay are generally handled with hands rather than tools during the preparatory stage, whereas tools are expected to be used more on the visible surface during the last stages of the carving process. We observe that *MCB* values corresponding to T_1_ are similar to values of impressions found in inner voids, thus suggesting that these toolmarks may be impressions of a smeared fingermark instead. However, our qualitative experiments (Fig. 4) show that smeared fingermarks are more imperceptible than brush strokes and display ridges of almost equal depth. Hence, we hypothesize that strokes T_1_ are generated by a tool like a brush with dry hair or a modeling tool rather than belonging to a smeared fingermark. Figure 4 also shows that marks from the toothed modeling tool are wide and of almost equal width. Impressions left by a paintbrush are thinner and denser, and corresponding ridge breadth varies according to the brush hair length. The differences between the mean ridge breadth (*MRB*) values calculated for 3D models and those measured in 2D images (table S4) are all lower than or equal to 0.05 mm except I_B_, I_F_, and I_H_, which we would expect because positioning the ruler perpendicular to the bundle of ridges, and tangent to the plane where these lay, appears challenging.

### Age estimates from ridge breadth values

**Fig. 5.**
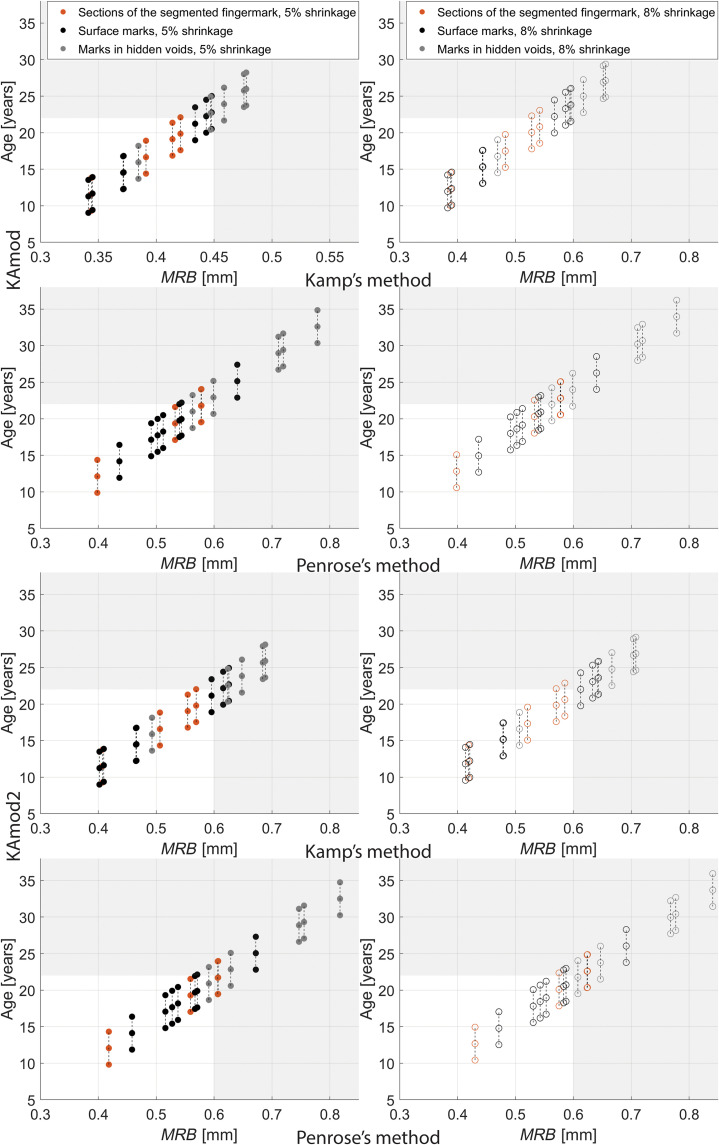
Plot of age estimates against ridge breadth values corresponding to preserved fingermarks. Results of age estimates from *MRB* values using the KAmod and KAmod2 regression models, separately shown for shrinkage correction and Kamp’s and Penrose’s definition. For each value, the minimum and maximum age is also represented (±2.25 years). In shadowed areas, we report age estimates for those *MRB* values that fall outside the empirical boundaries of data used to generate the linear regression model (*6*).

Figure 5 and table S2 show age estimates using the KAmod and KAmod2 regression models. We observe that age estimates from *MRB* values in Kamp’s definition are lower than Penrose’s, which we would expect because the *MRB* values in the former are on average lower than the latter. It is important to highlight a few aspects of age estimates from fingermarks. (i) Because ridge breadth mostly reflects body size at the time of growth, linear regression models are only valid during the period of (nearly) linear development until adult body size, which is around 22 years. (ii) In adulthood, ridge breadth does not vary considerably. (iii) The Kamp equation, which represents the reference model for later works, is determined on a population ranging in age from 4 to 22 years. (iv) The KAmod equation is restricted to the Czech population, which is made up of individuals who vary in body size, and individuals within the same population differ as well. (v) Intra-individual heterogeneity is present in epidermal ridge breadth. For these five reasons, we consider our age estimates to provide an indication of the age cluster to which the maker belongs, rather than the exact age they represent. After about 15 years of age, *MRB* values and age estimates become questionable, which is mostly due to shortcomings in the underlying modern demographic data rather than the computational method. Regarding age estimates using Kamp’s definition for either the KAmod and KAmod2 models, results are all above 15 years of age except those from A_4_, I_E_ for 5% correction, I_F_, and I_G_. Regarding age estimates using the Penrose’s definition for either the KAmod and KAmod2 models, results are all above 15 years except those from A_4_ and I_E_ (for 5% correction). Given that linear regression models are extrapolated on empirical boundaries for *MRB* values reported in Kamp *et al.* (*6*) (*MRB* = 0.3 to 0.6 mm, for ages between 4 and 22), age estimates calculated for *MRB* values that fall outside these limits are considered as proxies.

### Fingerprints and toolmarks under controlled settings

**Fig. 6.**
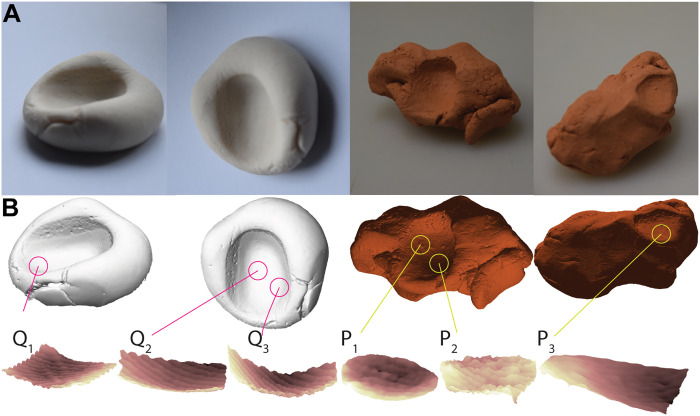
Quantitative experiment of fingerprints impressed under controlled settings. (**A**) Different views of a fingerprint impressed on fresh plasticine, and two fingerprints impressed on clay before firing. (**B**) Views of the corresponding 3D reconstructions. The regions of interest are displayed in circles. When the fingerprint is left with extreme care, impressions are clear and clay irregularities are almost imperceptible. On the contrary, impressions left without intention are thinner in depth and are interrupted by clay inhomogeneities.

Figure 6B shows 3D reconstructions of projection images representing fresh and fired clay items (Fig. 6A). For both objects, we repeat the procedure for mesh creation and ridge breadth determination used for the Rijksmuseum sculpture. First, each slice of the 3D stack is converted into a binary image and a corresponding 3D mesh is created. Each 3D mesh is then enhanced using surface fitting. Only one fingerprint is left on fresh clay, which is not fired, and two are left on clay, which is later fired. To showcase that ridge breadth values vary within the same fingerprint, we extract three sections from different locations of fingerprints and calculate ridge breadth on each: a straight line is traced three times in different locations, and we compute the average ridge breadth over the three runs. Because it is imprinted with care, the fingerprint left on fresh clay has clearer ridges and furrows (Q_1_, Q_2_, and Q_3_ in Fig. 6B). In contrast, fingerprints left accidentally on clay, which was later fired (P_1_, P_2_, and P_3_), exhibit more inconsistencies and local deformations. This underscores the same difficulties of examining fingermarks on terracotta sculptures experienced on the Rijksmuseum sculpture. *MRB* values produced using Kamp’s definition are always lower than those computed by Penrose for both clay pieces (table S3). As with the museum sculpture, low *MRB* values using Kamp’s definition represent the difficulties of tracing the line across ridges on the 3D-enhanced fingerprint without encountering clay inhomogeneities. Since impressions on fired clay are difficult to discriminate in several sections, the number of ridges in each run is less than four, while fingerprints on fresh clay have at least nine. It is important to note the variability in *MRB* values for both samples, depending on the section of the fingerprint: This implies that *MRB* values do not designate a person unequivocally but rather cluster the maker’s biological maturation. Furthermore, *MRB* values from both pieces tend to be similar, despite differences in age and sex. In the work of Králík and Novotný (*4*), the authors also note that the female participant who has spent her life working with hands, has coarser ridge breadths than most of the male participants. Here, the female volunteer is a professional ceramicist with many years of experience working with clay. Regarding the male volunteer, two of three sections display *MRB* values that are higher than 0.52 mm, which is the threshold above which *MRB* values for adult women no longer occur in the work of Králík and Novotný (*4*). The radial section (Q_2_) exhibits the highest observed *MRB* value (0.56 and 0.61 mm for Kamp’s and Penrose’s definition, respectively). When shrinkage is accounted for the fired piece of clay, the highest *MRB* values correspond to the P_1_ section. In terms of age estimation, KAmod and KAmod2 equations are only used for the fired piece of clay because shrinkage correction is not relevant to fresh clay, for which we use the Kamp model only. It is worth noting that the regression models used for age estimation are trained on a youthful population sample of European ancestry (<22 years old). As previously stated, linear regression algorithms are only valid for discriminating child makers from biologically adult makers. Both samples are biologically adult makers, according to Penrose’s definition of ridge breadth (table S3). The differences between these *MRB* values and those measured on 2D images (table S4) are all lower than or equal to 0.05 mm and the lowest refer to Q_1_, Q_2_, and Q_3._

## DISCUSSION

Here, we propose a 3D micro-CT acquisition method for partial preserved impressions left on a fired clay sculpture, as well as an interpretative framework to analyze such impressions. We select eight partial fingermarks on the visible surface, five in inner voids, and three toolmarks on the sculpture *Study for a Hovering Putto*, attributed to Laurent Delvaux. For each epidermal ridge impression and toolmark, we determine its 3D mesh from the corresponding 3D volume and enhance it to compute breadth and crease counts. We estimate the age group from finger ridge breadth using linear regression models based on fingermarks from volunteers of European ancestry described in the literature. When results are combined, values indicate adult maker(s). We compare ridge breadth and ridge count values of fingermarks against values of various toolmarks. Furthermore, we use qualitative experiments to demonstrate that narrow linear striations that resemble smeared fingerprints are most likely from a brush with thin hard hair or toothed modeling tool with thin teeth.

Most of the fingermarks found on the terracotta surface have ridge breadths that are greater than the reference value for male adults. Thinner fingermarks occur, and our hypothesis is that these are results of deformation pressure or impressions of proximal sections of a fingermark. The partial impressions found in inner voids display wider ridges that form slight “umbrella shapes” (*5*), which might be the result of wet fingers or a heavier pressure on fresh clay. Despite well-founded doubts on the analysis of small-scale and fragmentary fingermarks, several studies focus on similar impressions (*4*–*8*, *11*–*14*, *16*). From our results on the preserved fingermarks found on the sculpture, we notice that some ridge breadth values are similar, thus suggesting that certain fingers or finger portions are preferentially represented during a specific molding process. We cannot rule out the involvement of other makers in the preparation process solely based on the ridge breadth values: A single fingerprint displays variations in ridge breadth depending on the section considered, and similar ridge breadth values occur among fingerprints of different individuals, as shown in our experiments. Matching multiple fingermarks could help with clarification, but the extreme fragmentary nature of these marks makes matching impractical. However, given the trustworthiness of the record for the Delvaux sculpture at the Rijksmuseum (*17*), we are more inclined to believe that these are all the work of the same artist.

The size of a clay object, as well as impressions on its surface, changes during drying and firing. Ceramic linear shrinkage varies between 0 and 20% (*4*). Hence, biological variability of the ridge breadth as imprinted on terracotta is confounded by shrinkage and pressure deformation. Here, shrinkage values correspond to known reference numbers. Investigating the clay used to model the sculpture would lead to more precise shrinkage factor estimates and consequently, adjusted ridge breadth values and age estimates.

Most fingermark-based studies in archaeology use single- or dual-camera acquisitions, an external light source, and magnifying lenses. Calibration is performed in single-camera acquisitions using a square of calibrated paper placed near each fingermark parallel to its surface plane. By positioning fingermarks in the center of the image and taking care that each part of the mark is acquired in focus, lens distortion around the edges of the field of view is reduced. The camera is situated as perpendicular to the fingermark surface as possible. Hence, even though works in related fields use less sophisticated systems (*4*, *6*), authors’ settings minimize 2D camera distortions and produce sound results. Despite the usefulness of their results, such methodologies might exhibit errors when collecting images of fingermarks because of the difficulty of positioning the calipers (*6*) and camera, or any other combination of light source and camera. Because of the dimensions of the sculpture under investigation, precise positioning of fingermarks under a stereomicroscope is time-consuming and easily error prone. On the other hand, using 3D micro-CT results in a safe and reproducible method for objects with dimensions similar to those of the artwork under study, and the produced 3D models give a view-independent reconstruction of preserved fingermarks.

Future improvements on the experimental methods and processing of the 3D spatial information should be addressed. Regarding CT scanning, for example, despite the use of copper filters during acquisition, we still detect some beam hardening artifacts on 3D slices. To overcome this issue, we plan to use more filter layers made with copper or another material. In terms of analysis methods, instead of manually detecting outliers, automatic selection might be implemented, for instance using the Mahalanobis distance. Furthermore, for simplicity and storage savings, our computations are based on 3D surface meshes. Alternatively, 3D voxel-based analysis might be investigated. Inspired by the work of Galbally *et al.* (*18*), we remove the general shape of the mark to enhance high-frequency content. The main differences between our study and Galbally’s are the type of impressions studied and the method used to enhance the 3D data. We study fragmentary impressions left on irregular material and we use surface fitting. Because of local clay inhomogeneity, surface fitting results in local distortion, making the positioning of the origin and direction of a straight line challenging. Mineral grain size is frequently comparable to ridge dimensions, thus altering ridge borders. Moreover, a high-order polynomial is used to fit the surface. Alternative surface fitting approaches, which might be better suited for larger sections, and a comprehensive investigation of the introduced local distortion should be addressed in further development of the current work. In addition, future improvement should involve automatic selection of the line direction according to the orientation of ridges, for example, by selecting the direction perpendicular to ridge curvature vectors.

Estimating the age cluster of an artist can be useful in those cases where the master was closely working with pupils, and more information extracted from surviving marks can add value to artworks by supporting artistic attribution. There are concerns in the forensic community regarding some of the statements about marks on clay artifacts, such as the categorical attribution to fingers, sex, and age determination based on calculations of the ridge breadth and density. Surely, the presence of a clear general pattern can be a strong indicator that preserved epidermal ridge impressions belong to (a part of) a fingermark. Other parts of a hand or foot, however, exhibit patterns as well. Basically, determining whether an impression may be assigned to a finger, palm, or even a foot is heavily dependent on multiple factors, such as the quantity of the visible papillary lines and their quality (i.e., the level of detail). In forensic investigations, experts often encounter partial finger and palm marks. This means that in most cases, they do not observe all the pattern information of the possible source, be it a finger, palm, or even a foot. Nevertheless, such a partial mark can be analyzed or an estimation about the source can be made. When many marks are found adjacent to each other, forensic professionals can sometimes deduce which fingers or which (part of the) palm left the marks. Even in the event of a single mark, the quantity of the visible epidermal ridges, in combination with their quality, is extremely important for the level of confidence in estimating the likely source of the mark. Future efforts should focus on reaching a decision with the assistance of well-trained forensic experts. Hence, we believe that collaborative efforts among interdisciplinary experts are essential for successful fingermark selection, pattern recognition, and maker provenance.

## MATERIALS AND METHODS

This section focuses on the technology and methods used to study finger ridge impressions and strokes from serrated modeling tools left on a terracotta sculpture in 3D virtual space.

### Sculpture selection at the Rijksmuseum

We collaborate with the curator and conservator from the Conservation Department for Ceramics, Glass, and Stone at the Rijksmuseum on the selection of *Study for a Hovering Putto* (*17*), attributed to Laurent Delvaux. Following the paleodermatoglyphic approach of Králík and Novotný (*14*), we first identify the origin of an impression. Then, according to their conditions, we choose which impressions to study. We find several striations of varied widths from modeling tools, as well as incomplete friction ridge impressions, mostly on the top backside of the body. We select three strokes from modeling tools and eight epidermal ridge impressions on the visible surface of the upper part of the body. Furrow and ridge patterns can be found on human fingerprints, palms, knuckles, and even feet. We presume that the artist used fingertips to model the artwork for three main reasons. First, the dimensions of the sculpture are ideal to handle the object using fingertips. Second, the position of ridges and furrows on the sculpture suggests that fingertips were used at these locations. Third, we support the safe assumption that one sculptor would often use a minimum of six different fingers when modeling clay: thumbs, index, and middle fingers of both hands. Hence, we consider marks found on the outer surface as fingermarks throughout the manuscript. We choose an object with clear impressions of papillary ridges and toolmarks and made of terracotta because x-rays can penetrate the material and reveal inner structures in addition to surface information. In addition, it is crucial that the object’s size and shape fit inside the scanner cabinet: The sculpture is 14 cm wide, 8.5 cm deep, and 28.1 cm tall. Three additional fired clay putti in the Rijksmuseum collection are credited to the same artist. According to the records for the Delvaux sculptures at the Rijksmuseum (*17*), these four naked putti, which are in various positions and clinging to fragments of a drapery, must have belonged to a larger whole, probably a monument. Several limbs are missing. The bozzetti, which are rare in the Netherlands, remind of the putti on the epitaphs of Adriaen Vrijburgh and Ferdinand van den Eynde in the *S. Maria* *dell’Anima* in Rome, made in marble by Francois Duquesnoy (1597–1643). On the hovering putto, fingermarks are all fragmentary (the largest fingerprint detected is less than 1 cm^2^) and are mostly visible on areas of the surface that look incomplete or in regions that could only have been molded with bare hands, such as the shawl. There are no visible fingermarks on the face, front body, legs, or arms, which appear in finished sections. Preservation of ridges substantially varies from one part of the fingermark to another. Moreover, clay is usually not totally homogeneous over the surface due to the mineral particles within clay that could distort or disrupt ridge details. We acquire only well-preserved fingermarks, and we discard visibly deformed marks.

**Fig. 7.**
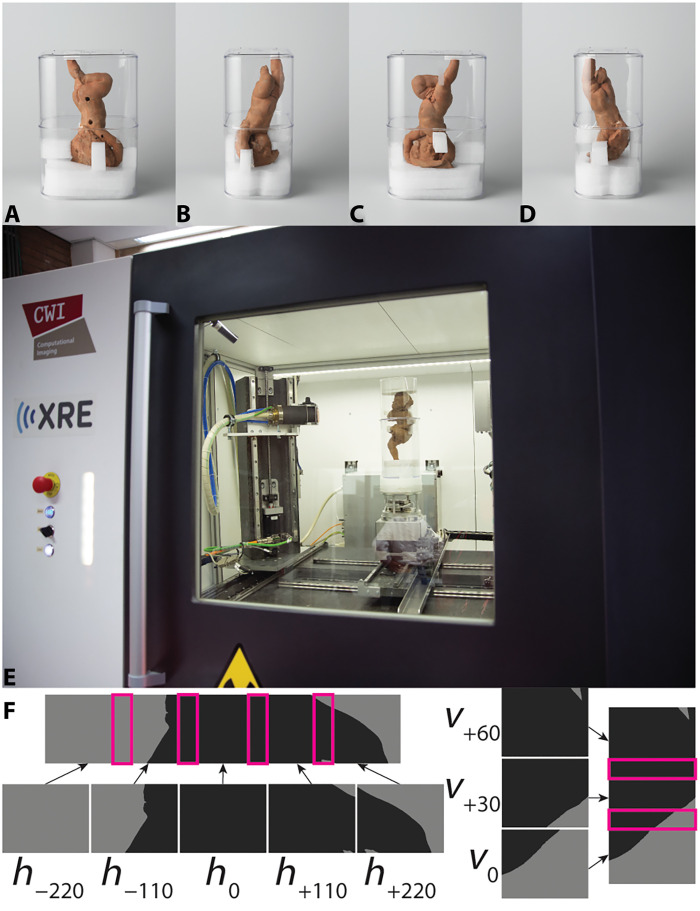
Schematic overview of the 3D micro-CT setup to acquire preserved impressions from a terracotta sculpture of the Rijksmuseum collection. Top row: four views of the artwork within the mounting support. (**A**) Three openings to facilitate attachment and a portion of the shawl. (**B**) Main lateral body facing the observer. (**C**) The face and upper shawl. (**D**) View opposite that of (B). (**E**) View of a test Rijksmuseum artwork and the bespoke mount inside the scanner cabinet (FleX-ray Lab, CWI). (**F**) (Left) Five horizontal images acquired during test acquisition. The parameters *h*_0_ and *v*_0_ refer to the reference horizontal and vertical detector positions, respectively. The detector is moved ±110 mm left/right relatively to *h*_0_, and +30 mm vertically from *v*_0_. (Right) Three vertical images are acquired during test acquisition phase. On a projection image, clay appears darker where the object is particularly thick (main body with the shawl). Feet, arms, and hair appear lighter.

We use a bespoke mount (Fig. 7, A to D) to make moving operations inside and outside the cabinet possible and to protect the sculpture’s fragility. Also, the object must be stable during CT scanning as any movement could cause unwanted artifacts on reconstruction slices. The support consists of a rectangular plastic container with rounded corners measuring 22 cm in width, 15 cm in depth, and 33 cm in height. Additional supports cut from polyethylene foam of various densities are placed around the head, shawl, and straight leg foot for more stability. Plastic is ideal for both the mounting support and foam because it is nearly transparent to x-rays compared to clay. The artwork is positioned vertically within the cabinet to achieve higher object magnification and consequently, higher resolution (Fig. 7E).

### CT settings

The general purpose of the FleX-ray Lab (Centrum Wiskunde & Informatica, Amsterdam) is to carry out experiments in the research domains of mathematics and computer science in an accessible and flexible manner (*19*, *20*). The scanner consists of a cone-beam microfocus x-ray point source (limited to 90 kV and 90 W) that projects polychromatic x-rays onto a flat panel detector with CsI(Tl) scintillator (1536 × 1944 pixels, each pixel is 74.8 μm × 74.8 μm). A rotation stage upon which the sample is mounted is located between the tube/source and detector. In our experimental setup, we place the object in such a way that the magnification factor leads to an effective voxel size of 25 μm in the reconstruction. We set the tube voltage at 90 kV and tube power to 50 W. For each projection, the exposure time is 400 ms. To reduce the effects of beam hardening, we mount combined copper filters of 0.15 mm thickness on top of the source. Before the acquisition starts, we draw a line crossing the object on the projection image to avoid zero photon counts occurring inside the object and to ensure that these photon counts do not exceed the limit counts for noise.

### Fingermark-based acquisitions

To reduce collection time and data storage, we CT scan only the whole segment of the sculpture that covers all the fingermarks. Before the scanning operation, we run a test acquisition to place the motors (tube, detector, and rotating stage) at the segment with fingermarks. Given that preserved fingermarks are thin, incomplete, and distorted, locating epidermal ridge impressions is challenging. The surface details leave relatively little contrast on x-ray projection images and identifying them on the outside surface using visible light is therefore easier. Hence, we attach a metal marker onto the plastic mounting support at the same height as the segments to identify fingermarks in projections. We can easily detect the metal pin on projection images and take this out before the scanning process begins.

Since the detector size is insufficient to catch the entire segment at the required resolution of 25 μm, we move motors horizontally and vertically as many times as necessary to fully capture the segment in a process known as tiling. During postprocessing, we stitch tiles together to produce the complete segment. One tile specifically refers to a stack of projections (.tif files) that are collected at certain source and detector coordinates. Each tile has 2401 projection images, meaning that projection images are acquired at 2401 evenly distributed angles around the object. We perform tiling by moving the source and detector around between scans, and a full rotation of the object is recorded for each location. For stitching to be successful, each tile must exhibit roughly a 20 to 30% overlap with adjacent tiles. By moving the source and detector to five different horizontal and three different vertical locations (Fig. 7F), a total of 15 tiles are produced.

Before each tile scan, a dark-field image is acquired: The tube is turned off to acquire an image of the detector offset count. After turning the tube back on, a flat-field image is acquired: A projection is recorded without the artwork in the field of view, giving an image of the beam profile. Following the tile scan, a second flat-field is recorded to correct for shadowing effects. Flat-field and dark-field images contain photon count data needed to preprocess images before the actual image reconstruction.

### Tile stitching and ROI CT reconstruction

In the postprocessing phase, we use FleXbox (*21*) and ASTRA Toolbox (*22*) to stitch the stack of tiles and compute a 3D reconstruction volume using the resulting stitched projections, respectively. We perform tile stitching and 3D CT reconstructions in Python version v3.6, ASTRA v2.1.0, and FleXbox v1.0.0. FleXbox is a lightweight Python-based CT reconstruction toolbox that provides easy access to its internal routines and in which ASTRA toolbox is integrated. In addition, it enables fast multi-GPU reconstructions on a single workstation. First, we join tiles horizontally, meaning that projections at equal angles are stitched together, resulting in one single stack of projections. Then, we merge the three resulting stacks of stitched horizontal projections, corresponding to three different vertical positions, to create a complete projection. Second, we reconstruct the obtained stack of projections onto a 3D volume. We reconstruct the volume at low resolution first because computing the full reconstruction at full size exceeds time and storage constraints. We then examine the obtained 3D stack of reconstructed slices to determine the coordinates of the ROI per fingermark and reconstruct this specific volume at full resolution. We save the reconstructed slices as .tif files, where each file is a grayscale intensity image. We manually remove the first and last slices of the stack, which usually contain primarily support material like plastic sponges.

### Image segmentation to 3D surface mesh modeling

We convert each grayscale intensity image into a binary image using Otsu’s (*23*) segmentation method (Fig. 1), which finds the threshold that minimizes the intraclass variance of the thresholded black and white pixels. We then convert the grayscale image into a binary one according to the computed threshold. In each grayscale intensity image, mineral grains appear as connected pixels of heterogeneous shape and dimension. Air is trapped between clay folds laid over each other before molding, and it appears darker than clay and mineral grains. In each binary image, clay and mineral grains are white pixels, while air within the artwork and on the background is black. We smooth the borders and eliminate small grain particles inside the area of interest. We then load the stack of binary images in Slicer3D (*24*), where the triangular surface mesh is generated, and we export to model file (Wavefront OBJ format).

### Section-based enhancement of impressions

We use MeshLab as 3D mesh editor to brush impressions of interest and to eliminate disconnected sections (*25*). Then, we import the 3D surface mesh in MATLAB R2022a for 3D cloud processing (Fig. 1), where we first manually detect and remove remaining points located outside the area of interest. In literature, given that a fingermark on a fired clay piece is frequently concave, the linear measure perpendicular to a bundle of ridges is more a tangent of the curve than the true length of the curve. For this reason, we enhance the 3D cloud to trace a straight line over ridges (using Kamp’s definition) and furrows (using Penrose’s definition). We consider the crease/furrow details as the high-frequency contents and the general shape of impressions as the low-frequency content. To remove the low-frequency content, we fit the 3D cloud with a high-order polynomial (i.e., we use “poly_55” from the function “fit” of the “Curve Fitting Toolbox” in MATLAB) and calculate the difference between the original cloud and the fitted surface. The epidermis is elastic and it becomes momentarily deformed when imprinted. Epidermal impressions on fresh clay are affected by the epidermal softness, clay hardness, and presence of water in the clay. Since these factors can cause slight or substantial changes even in local sections of a single impression, we split the largest partial fingermark into four different sections. We then fit a surface for each section separately. Moreover, given that fingermarks are reverse casts of actual marks, we work on the reverse of the actual 3D mesh when we study the creases.

### Calculation of ridge breadth and ridge density values from preserved fingermarks: Related works

According to research in archaeology, ridge breadth and ridge density values contribute to characterize the social and organizational structure of populations in past societies. Experts observe a high correlation between age and ridge breadth and they estimate the age of makers with sufficient accuracy even from incomplete fingermarks. Finger patterns are complete by the seventh month of natal development and they expand in size without additional topological alterations as hand size increases during growth. On average, variations in ridge breadth are on the order of 0.05 mm in a single hand and the largest variability in ridge breadth is attributed to age. In general, ridges on palms tend to be coarser than ridges on fingers. Ridges tend to be coarser on thumbs and finer on ring fingers. There are several methods used to determine the ridge breadth and ridge density values. Traditionally, scholars calculate the *MRB* on fingers to estimate the age. In particular, definitions from Penrose *et al.* (*26*) and Kamp *et al.* (*6*) are mainly used. The definition of Penrose is based on black-ink fingermarks on paper: They calculate the single ridge breadth value as the distance between centers of two consequent furrows, and the *MRB* for a single fingermark is the mean of all single measurements. The definition proposed by Kamp *et al.* is the most widely used: They measure the length of a mark, from the initiation of the first ridge until the end of the last furrow, and divide this distance by the number of ridge-furrow pairs. The *MRB* for a single mark is then computed across multiple ridge breadths. Moreover, Kamp *et al.* asked 101 individuals of European ancestry aged between 5 to 20 to leave their finger impressions on clay. The authors then determine a linear regression model

Kamp:Age(months)=614×MRB(mm)−112 (1)

The error rate is approximately 4.5 years for a single individual, and it is less than a year for a group of individuals. Using this model for figurines and ceramic vessels produced by the ancestral Puebloan group in Northern Arizona, the authors demonstrate that figurines were manufactured by children (*MRB* = 0.42 mm) and vessels by adults (*MRB* = 0.54 mm). Despite the usefulness of the method proposed by Kamp *et al.*, it exhibits a broad error range and it fails to discriminate the sex of potters. In addition, the distant genetic relatedness between the training and target groups might overestimate the age. To overcome the pitfalls of Kamp’s methodology, Králík and Novotný (*4*) asked three groups of volunteers of different age ranges and sex to leave their impressions on fresh clay. The authors then test several linear regression equations for age estimation and calculate the differences between the true and estimated age for each equation. First, the length of a bundle of ridges is measured on the longest possible section perpendicular to the ridges. For each measurement, the single ridge breadth is then calculated as the ratio between this length and the number of ridges. The mean value for each object, which corresponds to the *MRB* of a single individual, is determined from ridge breadth values of all fingermarks on that object. In their population sample, *MRB* < 0.39 mm corresponds to children and makers under 15 years of age, while *MRB* > 0.52 mm come solely from adult males. Since one of their equations, the KAmod formula (Eq. 2), is based directly on the data, they consider it the best equation for age determination

KAmod:Age(months)=[614×MRB(mm)]/(1−ts)−112 (2)

where *ts* is the true shrinkage. In general, the authors observe that age and sexual dimorphism can act together on ridge breadth variability for *MRB* > 0.4 mm. On the basis of experimental and forensic research, Fowler *et al.* (*5*) propose an interpretative framework to classify fingermarks. They determine a modified version of the KAmod regression equation for each individual mark, the KAmod2 equation

$$\mathrm{KAmod}2:\mathrm{Age}(\mathrm{months})=614\times MRB_{C}(\mathrm{mm})-112$$ (3)

where *MRB_C_* is the *MRB* value corrected for shrinkage

$$MRB_{C}=MRB_{O}+(MRB_{O}\times t_{s})$$ (4)

with *MRB_O_* being the observed *MRB*. The authors distinguish three groups: adult males (*MRB* > 0.52 mm), prepubescent children (*MRB* < 0.37 mm), and a third group that includes adolescents, adult women, and men. Ridge breadth values are calculated using both Kamp’s and Penrose’s definitions. Their analyses indicate that two-thirds of the fingermarks were likely from adult men and teenage boys and the remainder from adult women and adolescent girls. To generate a model to determine the sex, ridge density values are correlated with comparable data from populations with close ancestry. Their resulting model suggest that men have a greater than 95% probability of being identified with values <13 ridges/25 mm^2^ and women have the same probability of being identified with values >16 ridges/25 mm^2^. It is worth noting that these thresholds change according to the population under study. Moreover, ridge breadth and ridge density values may differ depending on the area of the fingertip considered (e.g., radial, proximal, and ulnar).

### Calculation of ridge breadth values and age estimation in this work

Here, ridge breadth is calculated using the methods of Kamp *et al.* (*6*) and Penrose *et al.* (*26*). Following Kamp’s definition, we determine the ridge breadth value as the ratio between the distance from the first to the last ridge and the ridge-furrow pairs. Following Penrose’s definition, we calculate the ridge breadth value as the distance between two centers of subsequent furrows. To account for intraobserver error, we run three different measurements on each fingermark. Multiple measurements refer to the same 3D model and do not include repeated CT scans of the object. For each measurement, the user selects the origin and direction of a straight line that intersects the bundle of ridges on the longest possible section perpendicular to this bundle. Once the line origin is selected, a circle centered at this origin assists the user in choosing the line direction. We then use the Möller-Trumbore algorithm to calculate the intersections between the line and the ridges (*27*). We determine the number of ridges as half the number of intersections. Regarding the shrinkage factor, we use shrinkage values corresponding to the terracotta used in Bernini's workshop (*10*). Hence, we correct each *MRB* value and age estimate for 5 to 8% shrinkage in Eqs. 2 and 3. To calculate the ridge count and compare this value between different impressions, we should consider standard areas of 25 or 6 mm^2^. However, impressions on terracotta are fragmented, and the use of a standard area of reference is unfeasible. Given that *MRB* specifically refers to the mean of epidermal ridge breadth values, we refer to *MCB* to welcome breadth values from creases left by epidermal impressions and modeling tools. In the Supplementary Materials, we add figs. S1 and S2 where crease breadth values are plotted separately for epidermal ridge impressions and toolmarks, respectively. In fig. S3, we plot results obtained under equal number of creases and crease-furrow pairs in each run. In table S4, we report measurements of ridge breadth on 2D images of each partial fingermark found on the sculpture, as well as each section of the fingerprints left by the volunteers (i.e., P_1_, P_2_, P_3,_ Q_1_, Q_2_, and Q_3_).

### Experiments with fresh clay under controlled settings

In the first experiment, we ask a trained professional ceramicist (>60 years old, female) and an untrained volunteer (26 years old, male) to leave their impressions on fresh clay. The dimensions of the clay objects are approximately 3 cm by 3 cm by 2 cm. The untrained volunteer leaves his fingerprint on fresh plasticine with the intention of impressing a clean mark. The trained ceramicist leaves several fingerprints unintentionally on another piece of clay, which is later fired at 900° to 950°C for approximately 12 hours. The piece of clay is first fired at 100°C/hour up to 750°C/hour, then at 900°C/hour for 1 hour, followed by a slow cooling for 3 to 4 hours. We then CT scan both objects at 70 kV and 50 W. The exposure time is 600 and 100 ms per projection for the fresh and fired object, respectively. The voxel size is 0.025 and 0.030 mm for the fresh and fired piece, respectively, and the total number of projections is 3601 for both. We implement mesh generation and ridge breadth determination following procedures performed for the Rijksmuseum sculpture. In the second experiment, we compare impressions from three different carving tools on pieces of fresh and high-fire clay: a toothed modeling tool with teeth of approximately equal width, a paintbrush with dry and unused hair, and a smeared fingerprint. The paintbrush is a proxy for a carving tool with thin teeth. Before leaving any impression, we water the piece of clay.
