# Supplementary material for: Artist profiling using micro-CT scanning of a Rijksmuseum terracotta sculpture
Source: Sci Adv. 2023 Sep 20;9(38):eadg6073. doi: 10.1126/sciadv.adg6073 (PMC10511186; doi:10.1126/sciadv.adg6073)
Supplement: Supplementary file 1 — Figs. S1 to S3 Legends for tables S1 to S4 [file sciadv.adg6073_sm.pdf]

Science Advances
AAAS

Supplementary Materials for

Artist profiling using micro-CT scanning of a Rijksmuseum terracotta sculpture

Dzemila Sero *et al.*

Corresponding author: Dzemila Sero, d.sero@rijksmuseum.nl, d.sero@cwi.nl

Sci. Adv. **9**, eadg6073 (2023)
DOI: 10.1126/sciadv.adg6073

The PDF file includes:

Figs. S1 to S3
Legends for tables S1 to S4

Other Supplementary Material for this manuscript includes the following:

Tables S1 to S4

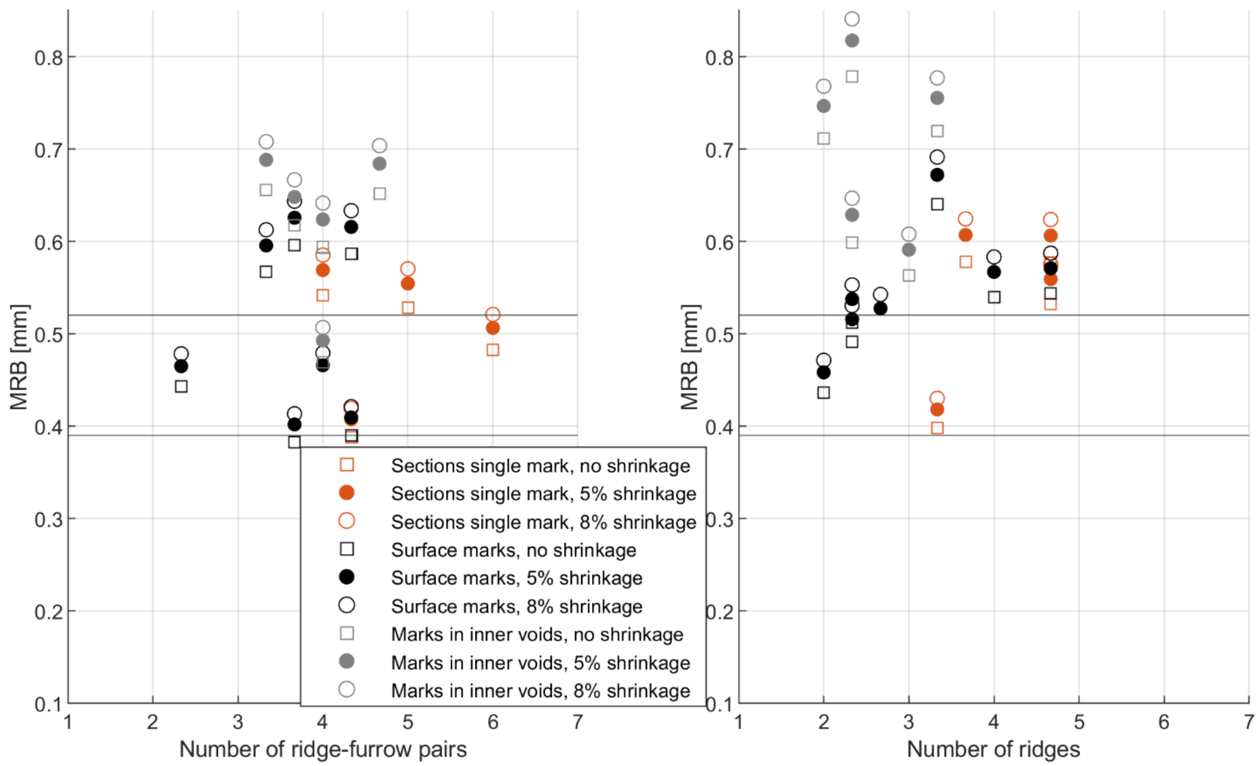

Fig. S1. Distribution of mean ridge breadth values corresponding to friction ridge impressions found on the Rijksmuseum terracotta sculpture. MRB values corresponding to eight visible marks and five marks in internal voids without shrinkage correction, and with 5% and 8% shrinkage correction, are plotted against the number of ridges. Sections of a single fingermark refer to the four sections of I_A. Each ridge breadth value is determined using the definition of Kamp (left) and Penrose (right). Horizontal lines at MRB = 0.39 mm and MRB = 0.52 mm refer to values found by Králík et al. (*4*) using fingerprints of European ancestry.

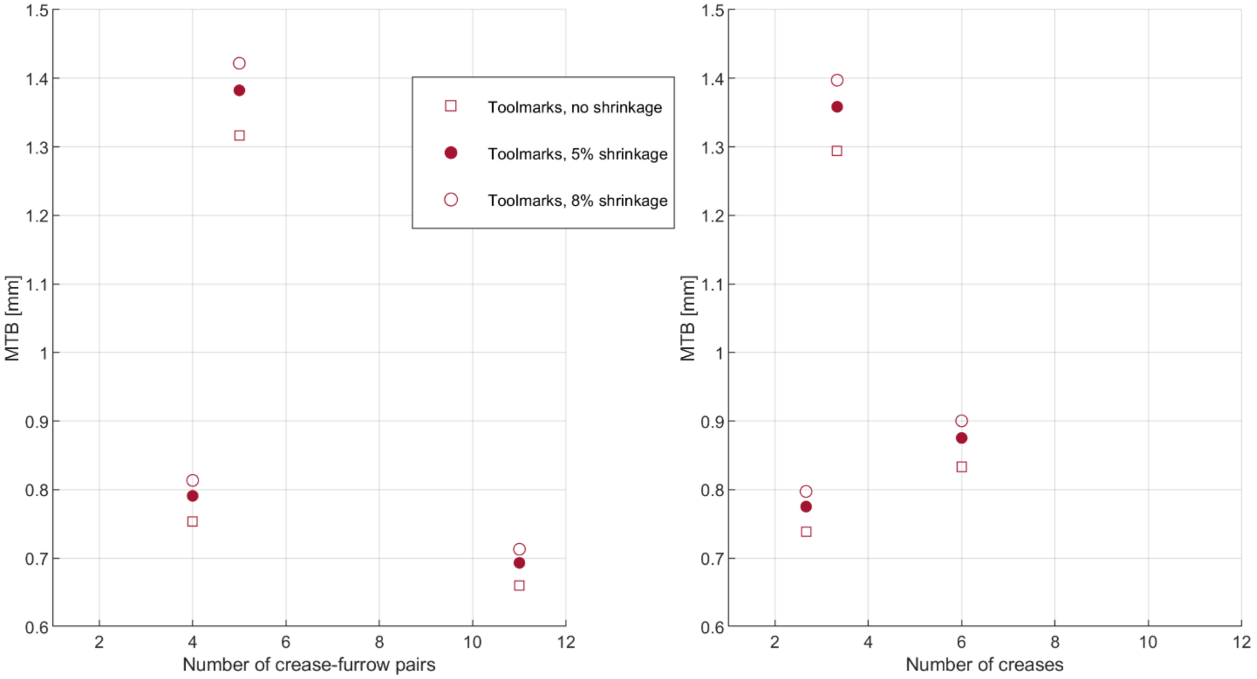

Fig. S2. Distribution of mean crease breadth values corresponding to toolmarks found on the Rijksmuseum terracotta sculpture. MTB (mean toolmark crease breadth) values corresponding to three toolmarks without shrinkage correction, and with 5% and 8% shrinkage correction, are plotted against the number of creases. Each crease breadth value is determined using the definition of Kamp (left) and Penrose (right).

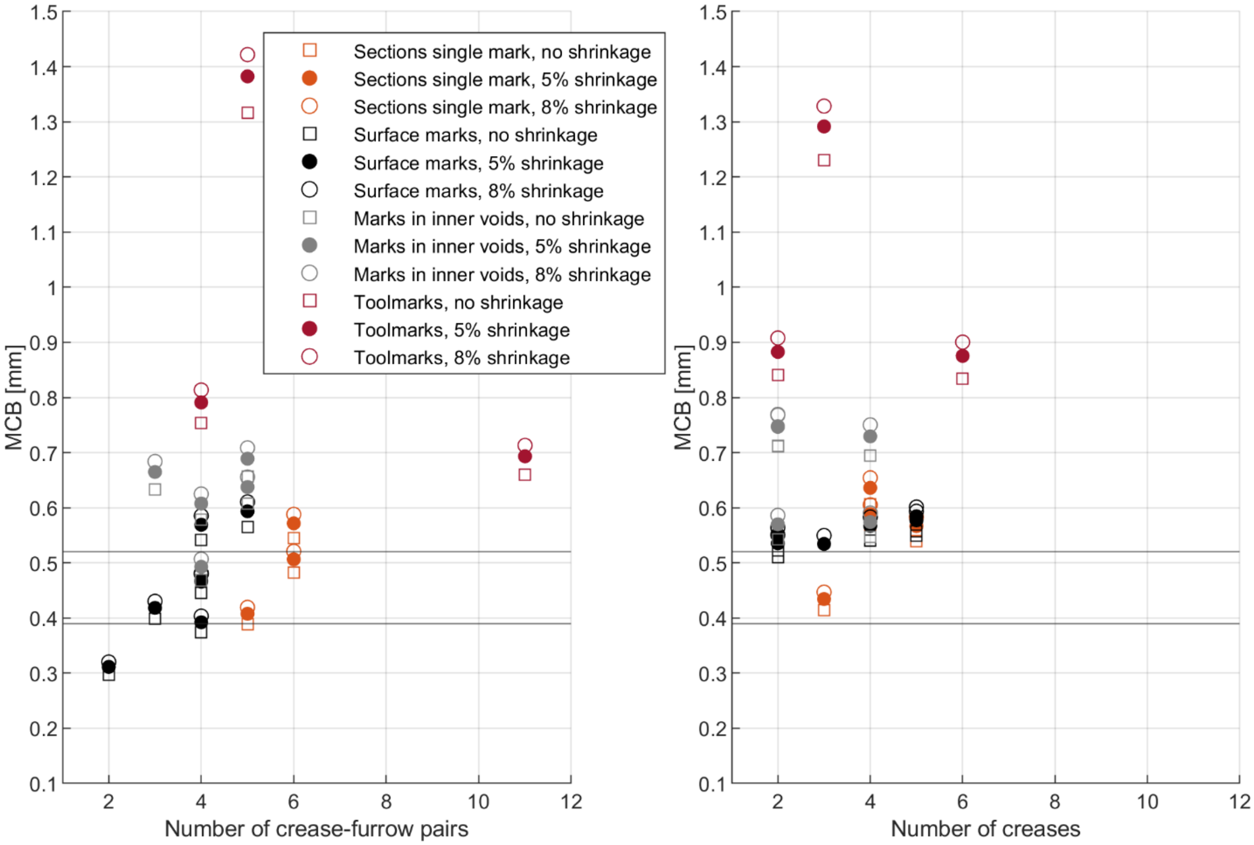

Fig. S3. Distribution of mean crease breadth values with equal number of creases and crease-furrow pairs for friction ridge impressions and two toolmarks. MCB values corresponding to each type of impression (eight visible fingermarks, five fingermarks in internal voids, three carving tools) without shrinkage correction, and with 5% and 8% shrinkage correction, are plotted against number of crease-furrow pairs (left) and number creases (right). Sections of a single fingermark refer to the four sections of I_A. Each ridge breadth value is determined using the definition of Kamp (left) and Penrose (right). Horizontal lines at MRB = 0.39 mm and MRB = 0.52 mm refer to values found by Králík et al. (*4*) using fingerprints of European ancestry. We keep the original number of creases only for one tool because its striations are dense and different in depth, thus making the selection of an equal number of creases difficult (T_1, MCB_O=0.66 mm with mean number of crease-furrow pairs=11 for Kamp, and MCB_O=0.83 mm with a mean number of creases=6 for Penrose).

Table S1. Numeric values of single crease breadth measurements for each impression found on the sculpture. Single measurements over three runs are reported for each impression found on the terracotta sculpture, following both the definition of Kamp as well as Penrose.

Table S2. Numeric values of age estimates from ridge breadth values corresponding to fingermarks. Age estimates (in years) are determined using the *KAmod* and *KAmod2* equations for each fingermark. Minimum and maximum age values are determined using ± 2.25 years. Age estimates for those MRB values that fall outside the empirical boundaries of data used to generate the linear regression model (*6*) are reported in italics.

Table S3. Age estimates and mean ridge breadth values corresponding to fingerprints impressed under controlled settings. MRB values (in mm) and age estimates (in years) are reported for fingerprints left on fresh and fired clay pieces. For the fired piece of clay (P_1, P_2, P_3), age is determined using the *KAmod* and *KAmod2* equations. For the fresh piece of clay (Q_1, Q_2, Q_3), only Kamp's regression model is applicable since shrinkage is not considered. Minimum and maximum age values are determined using ± 2.25 years.

Table S4. Single ridge breadth values measured on 2D images of surface fingermarks found on the terracotta sculpture and fingerprints left under controlled settings. Single measurements over three runs are reported for each surface fingermark found on the terracotta sculpture, as well as for each section of the fingerprints left by the volunteers under controlled settings. We measure ridge breadth following the definition of Kamp. The difference between the MRB values measured in 2D and those measured in 3D is computed. Differences in values above 0.05 mm are in italics.
